# Early, typical, and late talkers: an exploratory study on predictors of language development in the first two years of life

**DOI:** 10.12688/f1000research.145763.1

**Published:** 2024-07-16

**Authors:** Maria Grazia Logrieco, Ilaria Nicolì, Maria Spinelli, Francesca Lionetti, Giulio D'Urso, Giulia Carlotta Guerra, Valeria D'Aloia, Giusi Toto, Mirco Fasolo

**Affiliations:** 1 Department of Humanities, University of Foggia, Foggia, Apulia, Italy; 2 2Department of Neuroscience, Imaging and Clinical Sciences, Gabriele d'Annunzio University of Chieti and Pescara, Chieti, Abruzzo, Italy; 3 Department of Human, Legal and Economic Sciences, UNIDAV- Telematic University Leonardo da Vinci, Chieti, Abruzzo, Italy

**Keywords:** late talkers, early talkers, typical talkers, gender, language development, individual differences, interactive behaviors, gestures

## Abstract

**Background:**

The consensus in scientific literature is that each child undergoes a unique linguistic development path, albeit with shared developmental stages. Some children excel or lag behind their peers in language skills. Consequently, a key challenge in language acquisition research is pinpointing factors influencing individual differences in language development.

**Methods:**

We observed children longitudinally from 3 to 24 months of life to explore early predictors of vocabulary size. Based on the productive vocabulary size of children at 24 months, 30 children met our sample selection criteria: 10 late talkers and 10 early talkers, and we compared them with 10 typical talkers. We evaluated interactive behaviors at 3, 6, 9 and 12 months, considering vocal production, gaze at mother’s face, and gestural production during mother-child interactions, and we considered mothers’ report of children’s actions and gestures and receptive-vocabulary size at 15 and 18 months.

**Results:**

Results indicated early precursors of language outcome at 24 months identifiable as early as 3 months in vocal productions, 6 months for gaze at mother’s face and 12 months for gestural productions.

**Conclusions:**

Our research highlights both theoretical and practical implications. Theoretically, identifying the early indicators of belonging to the group of late or early talkers underscores the significant role of this developmental period for future studies. On a practical note, our findings emphasize the crucial need for early investigations to identify predictors of vocabulary development before the typical age at which lexical delay is identified.

## 1. Introduction

There is extraordinary variability in the vocabulary size of very young children ( Bates, Bretherton & Snyder, 1991; Fasolo, D’Odorico, Costantini & Cassibba, 2010; Fenson, Dale, Reznick, Bates, Thal, Pethick, 1994; Goldfield, 1987; Hampson & Nelson, 1993; Huttenlocher, Haight, Bryk, Seltzer & Lyons, 1991). The scientific literature converges on the notion that every child follows his own path in linguistic development while sharing the same developmental stages with others, with some children standing out as stronger or weaker in their language skills than their peers. Indeed, a child of 24 months acquiring English or Italian with a typical development has a mean of 300 words in production ( Brown, 1973; Caselli &-à Casadio, 2014), early talkers can have in their vocabulary up to 500 words at the same age, while late talkers tend to produce less than 50 words ( Rescorla, 2002). One of the major challenges in the study of language acquisition is therefore to identify factors that can contribute to individual variation in language development. Considering the complexity of language development which is the joint product of maturation and experience ( Carpenter, Nagell, Tomasello, Butterworth, & Moore, 1998; Tomasello, 1995) epidemiological studies have provided an integrative model of predictors. The model includes biological factors such as gen-der, child’s communication skills, and the quality of language stimulation a child receives from his parents as well as variables that may affect it, such as the level of education, and the socio-economic status ( Oller, Eilers, Neal & Schwartz, 1999; Stanton-Chapman, Chapman, Bainbridge & Scott, 2002; Zambrana, Pons, Eadie, & Ystrom, 2014; Reilly et al., 2007; Eadie, Levickis, McKean, Westrupp, Bavin, Ware, Gerner, Reilly, 2022; Hoff, 2013). While there are several studies on the effect of environmental factors on language acquisition, few studies have focused on specific child’s behaviors that are detectable from the early months. Considering the role of gender in language skills variability, literature states that girls have generally superior linguistic abilities compared to age-matched boys ( Zambrana et al., 2012; Huttenlocher et al., 1991). On the other hand, a recent meta-analysis pointed out that expressive language outcomes did not differ between boys and girls, although the variability among studies was significant ( Fisher, 2017; Wallentin, 2020). For what pertains to the child’s communicative characteristics, research in literature has stated that a combination of early neurobiological and genetic factors, such as gender, and concurrent environmental variables and individual characteristics, such as verbal and non-verbal behaviors may have an effect on the slow language development ( Schjølberg, Eadie, Zachrisson, Øyen, 2011; Zubrick, Taylor, Rice, Slegers, 2007) observable in the dyadic context already at 3 months. Indeed, longitudinal studies suggested that infant gaze at 10 months plays a quantifiable developmental role in children’s early linguistic and social–cognitive development ( Brooks & Meltzoff, 2015). In this direction, research on preverbal pro-ductions emphasized that canonical bubbling at 10 months represents an important predictor to identify infants at extreme risk for speech and language disorders at the end of the first year of life ( Oller, Eilers, Neal, and Schwartz, 1999). These studies provided important evidence on the investigation of early predictors of child later outcome. However, studies investigating predictors from the first few months onward use only parent report questionnaires to assess predictors while those using observational measures have considered language development starting from 10 months of age, thus, limiting our understanding of early candidate precursors. Following the multidimensional perspective suggested by prior works, in the present study we combined observational measures and parent report questionnaire, and we investigated the potential early markers of language development, including gender and communicative linguistic skills of the child arising from 3 months of age.

Therefore, in the present study we involved children in different point of the language spectrum – late, typical, and early talkers - as young as 3 months old, following them up to 24 months of age. Specifically, the aim of our research is to explore if different social cues ( Hollich, Hirsh-Pasek, Golinkoff, Brand, Brown, Chung, and Bloom, 2000) produced by the child since 3 months may be informative of the subsequent language outcome, that is the breadth of the productive vocabulary size at 2 years. Specifically, as social cues, we considered vocal production, gaze at mother’s face, actions and gestures, and receptive vocabulary size which represent the foundations for the later development of language (see McCathren, Yoder & Warren, 1999; Vihman, Macken, Miller, Simmons & Miller, 1985; Brooks & Meltzoff, 2005, 2008; Carpenter, Nagell, Tomasello, Butterworth & Moore, 1998; Bates, Thal & Marchman, 1991; Iverson & Thelen, 1999; Lyytinen, Eklund & Lyytinen, 2005; Thal, Marchman & Tomblin, 2013; Goldin-Meadow and Alibali, 2013). In addition, the role of gender was considered to potentially control for gender differences while exploring its contribution at the same time.

### 1.1 Late and early talkers

Late talkers are children who show a vocabulary delay in the absence of any sensory, cognitive, or neurological deficit ( Buschmann, Jooss, Rupp, Dockter, Blaschtikowitz, Heggen & Pietz, 2008; Rescorla & Lee, 2000; Silva, 1980; Whitehurst & Fischel, 1994). For the aim of the present study, we adopted the most common criteria found in the literature and focused on 24 months children who have barely reached a 50-word vocabulary and/or use no word combinations ( Fenson, Dale, Reznick, Thal, Bates, Hartung & Reilly, 1994; Dollaghan, 2013; Rescorla, 2002). It is well known that late talkers are not a homogenous group in terms of their developmental outcomes: some catch up, as captured by the “late bloomers” metaphor and some will experience persistent learning difficulties ( Chilosi et al., 2019). Furthermore, language delay has a multi-dimensional impact on learning (see, for example, Paul et al., 1997; Rescorla, 2002, 2005, 2009; Rescorla & Dale 2013) and socioemotional development (see, for example, Paul, 1991; Carson, Klee, Carson & Hime, 1997, 1998; Irwin, Carter & Briggs-Gowan, 2002). Hence, the early investigation of factors associated with a language delay is crucial for the early intervention so that education pro-grams can be implemented to maximize positive child developmental outcomes across a variety of domains (see, for example, Buschmann et al., 2015; Roberts & Kaiser, 2015; St Clair et al., 2011). Although there is a significant interstudy variability in the analysis of language development in late talkers ( Fisher, 2017), research findings have been able to identify a series of specific communicative and linguistic characteristics of late talkers. More specifically, different authors have shown that late talkers have a narrow phonological inventory of both vowels and consonants, mostly characterized by simple syllabic structures, unlike typical talkers of the same age ( Paul & Jennings, 1992; Rescorla & Ratner, 1996; Mirak & Rescorla, 1998; Carson et al., 2003). Interestingly, differences between late and typical talkers are not strictly limited to linguistic variables. In fact, empirical studies have suggested that some communicative behaviors of late talkers, such as the different degree in gestural production, represented potential early predictors of delay in subsequent language development ( Ellis & Thal, 2008; Thal et al., 2013). Studies on gestural communication in children with linguistic delay highlighted significant differences at 20 months between late and typical talkers, resulting in a disadvantage of late talkers in the production of gestures and the combination of gesture and gaze at mother’s face ( Fasolo & D’Odorico, 2002). In the same perspective, we aim to explore the communicative behaviors of late and typical talkers, but investigating instead, the predictors of language development from the first months of life. Moreover, we do not limit our examination to typical vs. late talkers, as traditionally done in the extant literature. Indeed, with the aim of better identifying what language development predicts more broadly, we involve into our analysis early talkers too. In terms of productive vocabulary, early talkers are children who scored around the 90th percentile based on parent report of toddler’s expressive language ( Fenson et al., 1994). Research has shown that early spoken language development can play a substantial role in oral and written language performance ( Lyytinen, Poikkeus, Laakso, Eklund & Lyytinen, 2001; Lyytinen, Ahonen, Eklund, Guttorm, Kulju, Laakso & Viholainen, 2004a, b; Scarborough and Dobrich, 1990). Indeed, there are several studies on the early talkers regarding their learning outcomes during school-age years (see, for example, Crain-Thoreson & Dale 1992; Lyytinen et al., 2001, 2004a, b; Scarborough and Dobrich, 1990). However, to the best of our knowledge, no study has investigated the communicative behaviors of early talkers at the initial stage of language development.

### 1.2 The present study

To sum up, there has been an increase in studies interested in identifying communicative and linguistic factors related to the variability of the child’s expressive language. However, most of these studies involved children older than 10 months of age. Based on the literature we have identified two categories of predictors that can be fruitfully investigated starting from the first months of life and can be considered key competences underlying the subsequent productive vocabulary size ( McCathren, Yoder & Warren, 1999; Vihman, Macken, Miller, Simmons & Miller, 1985; Brooks & Meltzoff, 2005, 2008; Carpenter, Nagell, Tomasello, Butterworth & Moore, 1998; Bates, Thal & Marchman, 1991; Iverson & Thelen, 1999; Lyytinen, Eklund & Lyytinen, 2005; Thal, Marchman & Tomblin, 2013; Goldin-Meadow and Alibali, 2013). With direct observational measures we explored the number of vocal behaviors, gaze at mother’s face and gestures produced by the child in interaction with the caregiver. Moreover, we evaluated actions, gestures and the receptive vocabulary size using the “Primo Vocabolario del Bambino (PVB)” questionnaire ( Caselli, Bello, Rinaldi, Stefanini, & Pasqualetti, 2015) that is the Italian version of the MacArthur-Bates CDI ( Fenson, Marchman, Thal, Dale, Reznick, Bates, 2007) filled out by parents. We have defined these predictors as interactive behaviors, as all these variables emerged in the social scene between child and adult ( Tomasello, 1995). Besides, we considered the role of gender to control for its potential effect, while also exploring its interplay and contribution with the set of interactive behaviors considered. Given the relatively small sample size and the explorative nature of the current study, we adopted a descriptive approach. We aim to investigate, between the 3rd and the 18th month of age, which interactive behaviors best predicted the probability to be at 24 months old a typical, late, or an early talker, and at which time point this may occur. If early intervention is to be provided in the critical period for language development, it is necessary to investigate and identify early predictors in younger children as early as possible. Hence the goal of the present study is to add information on an evolutionary period sparsely considered in the extant literature, and that is the study of developmental trajectories of early and late talkers starting from early months.

## 2. Methods

### 2.1 Participants

This study included 30 monolingual Italian mother–child dyads. The dyads were selected from a larger and longitudinal study on parenting and infant development in Italy at University of Chieti before the lockdown due to Covid-19. The vocabularies of 10 late talkers (LT), 10 typical talkers (TT) and 10 early talkers (ET) were selected out of a pool of 80 MB-CDI forms ( Caselli, Bello, Rinaldi, Stefanini, & Pasqualetti, 2015) completed by parents for children (42 males and 38 females) aged 24 months. Specifically:

- LT (9 males and 1 female) produced less than 50 words (M = 37; range: 15-59), corresponding to the 10th percentile of productive vocabulary size,
- ET (2 males and 8 females) produced more than 500 words (M = 562, range: 509-640), corresponding to the 90th percentile of productive vocabulary size,
- TT (4 males and 6 females) produced around 225 words (M = 290, range: 222-350), and their vocabulary size was closer to the 50th normal mean value.

Mother’s mean age was 34.20 (SD = 5.35). Concerning socioeconomic status, 80 % of them had a medium economic status, and 20% had a low economic income (LT: 70% medium income; ET: 80% medium income; TT: 90% medium income). In relation to educational level, 66.6% of mothers had a high school degree or less, and 33.3% a bachelor’s or master’s degree (LT: bachelor’s degree 40%, high school degree 50%, secondary school degree 10%; ET: bachelor’s degree 30%, high school degree 60%, secondary school degree 10%; TT: bachelor’s degree 30%, high school degree 70%).

### 2.2 Procedure

Participants were recruited at an Italian urban hospital within 2 days from the child’s birth, and mothers were asked to join a research project on infant development. The mothers who agreed to be part of the research study were then contacted 1 to 2 months later and invited to come to the laboratory of Developmental Psychology. Only mothers gave us their willingness to participate to the experiment in lab. A written informed consent (approved by the ethical committee) for participation in the study has been obtained by the mothers. Before testing, each mother–child dyads participated in a familiarization phase in a room next to the proper observational room, equipped with age-specific toys. The mother-child dyads were observed during a video-recorded semi-structured play session conducted in the laboratory when the child was 3, 6, 9 and 12 months old. At 3 and 6 months, the mother and the infant were observed during a video-recorded interaction session. The mother sat in a straight-backed chair without arms and was asked to “play and talk to your baby as you would at home”. The child was in front of the mother sitting on a bouncer at 3 months and on a highchair at 6. The sessions were videotaped. The camera was controlled by an experimenter present in the room and placed behind the mother. It framed both the infant’s face and mother’s face, thanks to a mirror positioned behind the infant’s station. The total session lasted approximately 4 minutes. At 9 and 12 months, mother and child were filmed while playing with a standard set of toys. The age-appropriate toy set represented objects with which all children were familiar in their everyday routines and were provided with the specific aim of stimulating communicative and interactive exchanges. At 9 months, at the beginning of the interaction, we provided cubes to build a tower, while in the middle of the interaction we provide a book with illustrations of animals. At 12 months, we provided an illustrated booklet with figures of animals and a set of toy food. The sessions were videotaped. The camera was controlled by an experimenter present in the room with a camera placed in front of the mother and the child. The interaction lasted approximately 6 minutes.

At 15-, 18- and 24-months, the parents filled the questionnaire MB-CDI. The “Word and Gesture – WG” version was administered at 15 months, while the “Words and Sentences – WS” version at 18 and 24 months.

### 2.3 Measures

2.3.1 Interactive behaviors in the first year of life

Two trained transcribers, working independently one from the other and using CHAT (CHILDES system) format ( MacWhinney, 2000), transcribed all the interactive behaviors at 3, 6, 9 and 12 months directly from the mother – child recorded interaction. The second transcriber was not informed of the subject’s group assignment until the transcription was completed. The interactive behaviors were coded with a multi-dimensional schema:

Vocal productions. Each production was counted as a single unit of analysis when separated by at least one second. Crying vocalizations and sounds of discomfort were not considered. The frequency of the child’s vocal productions per minute was calculated. Child’s vocal productions were coded as follows: vocalization (vowel sound typically produced open-mouthed), grunt (consonant sound produced by closed or semi closed mouth), onomatopoeia (sounds that reproduce the sound of animals, or the noise produced by vehicles or actions), babbling (syllabic sound consisting of a consonant and a vowel), proto words (sounds similar to words that take on specific meaning in certain contexts).

Gaze at mother’s face. The unit of analysis corresponds to a child’s gaze at mother’s face. Every time that the child looked at the mother’s face, the gaze was coded. Gaze at the mother’s body, hair, hands, and gaze to environment was not considered. The frequency of child’s gaze at mother’s face per minute was calculated.

Gestural productions. The unit of analysis corresponded to a child gestural production. Every gesture accompanied by other behaviors that signal the communicative intent (i.e., direct gaze to the interlocutor) was coded. The frequency of child gestural productions per minute was calculated. Gestural productions included deictic gestures: pointing (extending the index finger in the direction of an object, an event, or a person), giving (put an object towards the hand of the interlocutor), showing (put the object along the line of sight of the interlocutor). Moreover, we included representative gestures: iconic gestures (gestures which refer to objects, persons, or events, reproducing physical or functional characteristics), conventional (gestures with a culturally defined meaning and form e.g., waving bye, asking for silence pressing the index finger against the lips).

2.3.2 Reliability

Transcription reliability was calculated on 25% of the total videos. Point-by-point interactive behaviors comparisons were made, and the intercoder reliability ranged from 0,80 to 0,95. To maintain data consistency, only the material produced by the first transcriber was used for the final analysis. Inter-rater reliability for each predictor was computed by having a second rater that independently scored the subject performance on each measure. The inter-rater reliability was coded on 10% of the sample; the inter-coder reliability ranged from 0.83 to 0.94. Disagreements were solved with discussion to identify a unique score.

2.3.3 Interactive behaviors in the second year of life

The interactive behaviors in the second year of life at 15-, 18- and 24-months were assessed using the scores assigned by parents at the CDI. Specifically, we considered:

CDI Actions and Gestures. We considered the number of actions and gestures produced by the child reported by the mother at 15 months. This category included communicative gestures, games and routines, actions with objects, symbolic game (i.e., pretending to be mom or dad, imitating the actions of the adult, and playing pretend with objects). Data was missing for a male in the LT group for CDI actions at 15 months.

CDI Productive and Receptive Vocabulary Size. We considered the receptive vocabulary size of the child (the number of words understood by the child) as reported by the mother at 15 and 18 months. At 24 months, we considered the productive vocabulary size (the number of words produced by the child) which was used as a criterion for inclusion in one of the 3 groups (late, typical, and early talkers). The CDI Receptive vocabulary measure at 18 months was missing for two children in the LTs group (1 male, 1 female) and one female in the ETs group.

### 2.4 Analytic plan

**Figure 1.**
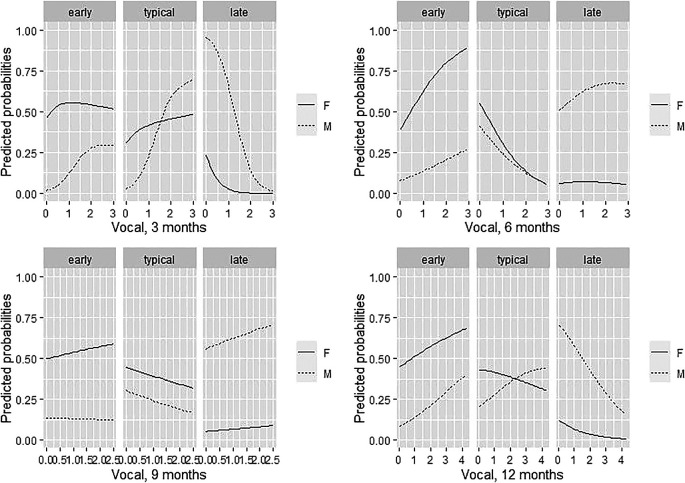
Predicted vocal production probabilities for males and females at 3, 6, 9, and 12 months, plotted against vocal frequency.

**Figure 2.**
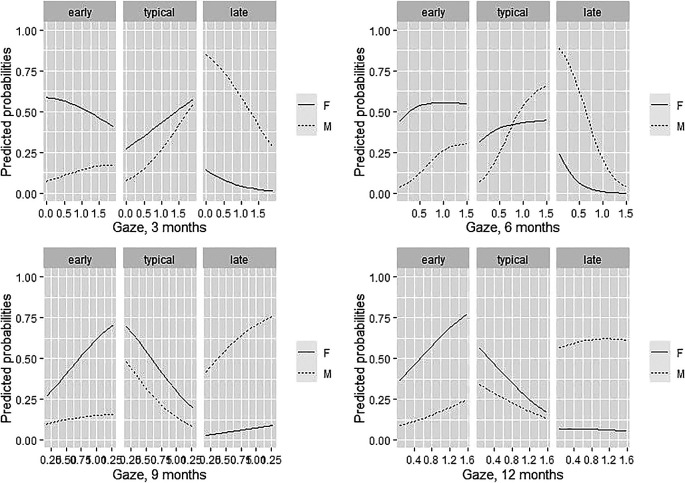
Predicted gaze probabilities at mother's face for males and females at 3, 6, 9, and 12 months, against gaze frequency.

**Figure 3.**
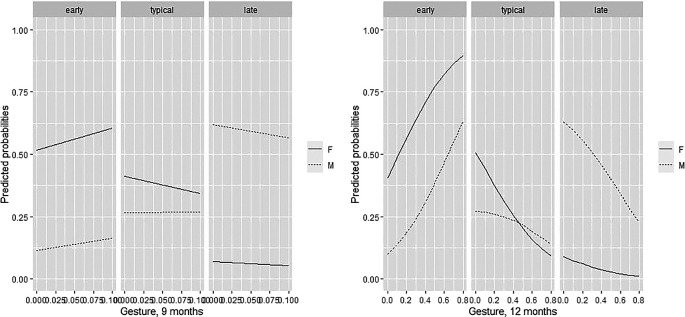
Predicted gestural production probabilities for males and females at 9 and 12 months, plotted against gestural frequency.

**Figure 4.**
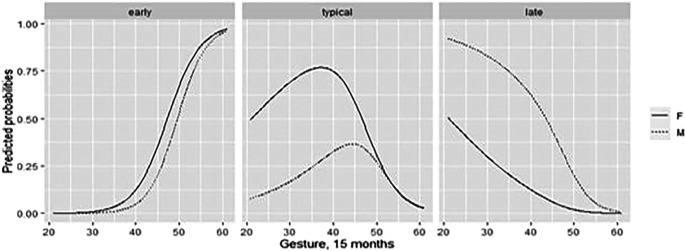
Predicted CDI action and gesture probabilities at 15 months for males and females, plotted against count.

**Figure 5.**
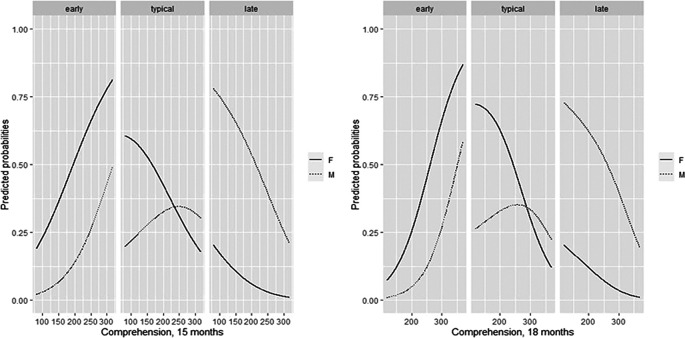
Predicted CDI receptive-vocabulary probabilities at 15 and 18 months for males and females, against count.

For the analysis of data, given the relatively small sample size, we avoided adopting a Null-Hypothesis Significance Testing Approach, rather we commented on the effect sizes describing patterns of findings ( Wasserstein et al., 2019). After having explored mean and standard deviation values as well as bivariate correlations among study variables, we ran and compared a series of multinomial logistic regression models including group membership (early, typical, and late talkers) as the outcome variable, and as predictors vocal production, gaze at mother’s face, gesture production, CDI actions and gestures, and CDI receptive vocabulary at the different time points investigated. We included one predictor at time in the regression models, to explore the differential effect of each variable across the different time points considered. As vocal production, gaze at mother’s face and gesture production were collected across multiple time points, we used Akaike Weights to identify what time point was more relevant for each specific variable. Akaike Weights represent a measure of the strength of adaptation for each model and the probability, conditioned to the set of models considered, that the same model can predict new data ( McElreath, 2016; Vandekerckhove Matzke & Wagenmakers, 2015; Wagenmakers & Farrell, 2004). Akaike weights range from 0 to 1 and the higher the value, the more the model comparatively describes the data accurately. In each multinomial regression model, as groups were unbalanced regarding gender, we included gender as a predictor to simultaneously control for its impact as well as exploring its contribution. As the sample was relatively homogenous in terms of socio-economic and educational background, we did not include any other control variable in the model. Linear predicting probabilities of belonging to a specific group as a function of the predictors considered at different time points were plotted and graphically explored ( Figure 1- 5). Finally, as a follow-up analysis, for the best model selected we provided the probability of belong to a specific group for low (< 25°), medium (< 25° < 75°) and high (> 75°) levels of the predictor. All analyses were run with the statistical software R version 4.2.3, package ‘nnet ( Ripley, Venables & Ripley, 2016) for multinomial regression analysis and gglpot2 ( Wickham, 2016) for the graphical representation of findings.

## 3. Results

### 3.1 Preliminary analyses

Means, SDs, and correlation values among variables of interest are reported in Table 1 and Table 2.

**Table 1.** Descriptive statistics.

		ET		TT		LT	
	Timing	Mean	SD	Mean	SD	Mean	SD
Vocal production	3 m	1.04	0.74	1.20	0.89	0.76	0.37
	6 m	0.87	0.72	0.53	0.51	0.93	0.80
	9 m	0.84	0.72	0.72	0.50	0.69	0.63
	12 m	1.50	1.28	1.16	1.25	0.76	0.54
Gaze at mother’s face	3 m	0.80	0.51	1.02	0.58	0.76	0.52
	6 m	0.61	0.33	0.65	0.33	0.40	0.23
	9 m	0.82	0.31	0.65	0.20	0.72	0.35
	12 m	0.71	0.44	0.53	0.39	0.58	0.23
Gestural production	9 m	0.02	0.06	0.02	0.04	0.03	0.06
	12 m	0.21	0.27	0.09	0.10	0.07	0.09
CDI action and gestures	15 m	52.1	5.99	42	4.47	37.66	9.44
CDI receptive-vocabulary size	15 m	232	67	187	65	161	59
	18 m	294	61	233	69	207	69

**Table 2.** Bivariate correlations (Pearson’s r) between study’s communicative variables.

	Voc 3 m	Gaze 6 m	Voc 6 m	Gaze 9 m	Voc 9 m	Gestures 9 m	Gaze 12 m	Voc 12 m	Gestures 12 m	CDI-A&G 15 m	CDI-RV 15 m	CDI-RV 18 m	CDI-PV 24 m
Gaze 3 m	.381	.239	-.150	.107	-.095	-.046	.201	-.186	.145	.118	.177	.371	-.011
Voc. 3 m		.309	.301	.113	.368	.257	.094	.015	.272	.046	.147	.222	.189
Gaze 6 m			-.108	.390	-.175	.525	.102	-.054	.436	.031	.236	.140	.252
Voc. 6 m				-.057	.388	-.026	-.080	.133	-.052	.027	-.053	-.228	.052
Gaze 9 m					.090	.024	.219	-.017	.413	.024	.100	.112	.061
Voc. 9 m						.147	.092	.350	.090	.296	.204	.052	.153
Gestures 9 m							-.049	-.140	.379	.072	.291	.234	.116
Gaze 12 m								.027	.097	-.023	-.108	.062	.175
Vocal 12 m									-.130	.240	.071	.039	.304
Gestures 12 m										.301	.341	.260	.293
CDI-A&G 15 m											.739	.638	.667
CDI-RV 15 m												.715	.418
CDI -RV 18 m													.487

### 3.2 Multinomial regression models

Akaike Weights derived from the comparison of multinomial regression models, as reported in Table 3, suggested that early vocal production at 3 months of age was more able to predict subsequent 24 months group membership than vocal production at 6, 9, and 12 months. In relation to gaze at mother, the model receiving most support was the one including gaze at 6 months, suggesting that gaze at the mother in this specific developmental period was more predictive of language development than not gaze at 3, 9, and 12 months of age. Regarding the gestural production, its assessment at 12 months was more predictive of group membership than gestural production at 9 months. Hence, vocal production at 3 months, gaze at mother at 6 months, gestural production at 12 months comparatively emerged as the most informative markers of later vocabulary productions, and hence group membership, at 2 years of age. A more in-depth analyses of findings for each predictor is reported in the paragraphs below.

**Table 3.** AIC and Akaike Weights.

Predictors	AIC	Akaike weights
Vocal production		
3 m	**60.74**	. **74**
6 m	64.19	.13
9 m	66.13	.05
12 m	65.26	.08
Gaze at mother’s face		
3 m	63.85	.20
6 m	**61.88**	**.54**
9 m	64.34	.16
12 m	65.30	.10
Gestural production		
9 m	66.	.34
12 m	**64.63**	**.66**

Note: In bold are models that received the best support in the different timing considered.

3.2.1 The role of child vocal production

As evidenced above, vocal production at 3 months of age was more predictive of group membership at 24 months compared to vocal production at months 6, 9 and 12. This result suggests that vocal productions that occur early in life are potentially first key predictors of verbal productivity at 24 months. More specifically, the graphical exploration of findings (see Figure 1) showed that the higher the vocal production at 3 months of age, the lower was the probability of belong to the LTs group with a stronger effect for males (see Figure 1) – which were still predominant in the LTs group. Predictive probabilities of belonging to each group for low (below the 25th percentile), medium (between the 25th and 75th percentile) and high (above the 75th percentile) levels of vocal production at 3 months ( Table 4). The pattern identified at 3 months of age was comparable to that at 12 months, though – coherently with the fact that this model was less supported by Akaike weights - the effect was less strong. At 9 months of age, vocal production was not predictive of group membership while at 6 months the higher the vocal production, the higher the probability for females to be in the ET group. No predictive effect for LT belonging was evident.

**Table 4.** The probability of belonging to the early, typical al late talkers for low, medium, and high values of the variable vocal productions at 3 months.

	Early talkers	Typical talkers	Late talkers
Values of vocal productions < 25 ^th^ percentile			
Female	0.30	0.45	0.23
Male	0.02	0.01	0.95
Values of vocal productions 25 ^th^-75 ^th^ percentile			
Female	0.38	0.53	0.08
Male	0.08	0.04	0.86
Values of vocal productions > 75 ^th^ percentile			
Female	0.43	0.55	0.01
Male	0.64	0.29	0.06

3.2.2 The role of gaze at mother’s face

Concerning the role of gaze at mother Akaike weights ( Table 3) suggested that a critical time point was 6 months, as depicted in Figure 2. Predicting probabilities of group membership for low, medium, and high values of gaze at mother’s face at 6 months are reported in the supplementary material. As it was for vocal reactivity at 3 months, the higher the number of gazes at mother at 6 months of age, the lower was the probability of belonging to the LTs group with the strongest effect for males. A comparable pattern emerged at 3 months, though with a less strong effect. At 9 and 12 months, gaze at mother’s face seems progressively less significant in predicting group membership, for both males and females. Hence, results suggest that gaze at mother at 6 months of age comparatively represents a better predictor (compared to gaze at mother at the other investigated time points) of language productivity at 24 months, and it might particularly help in identifying males that are more likely to become late talkers. The probability values of belonging to a group was then explored for the low, medium, and high values of the gaze at mother’s face at 6 months ( Table 5).

**Table 5.** The probability of belonging to the early, typical al late talkers for low, medium, and high values of the variable gaze at mother’s face at 6 months.

	Early talkers	Typical talkers	Late talkers
Values of gaze < 25 ^th^ percentile			
Female	0.31	0.44	0.24
Male	0.07	0.38	0.88
Values of gaze 25 ^th^-75 ^th^ percentile			
Female	0.37	0.51	0.10
Male	0.16	0.08	0.74
Values of gaze > 75 ^th^ percentile			
Female	0.41	0.55	0.03
Male	0.35	0.17	0.46

3.3.3 The role of child gestural production

For what pertains to gestural production, at 9 months few frequencies other than 0 emerged in the production of gestures, and the frequency per minute of gestures at 9 months seems to be relatively insignificant in predicting the group membership. A key time point for the prediction of group membership was age 12 months (see Table 3 and Figure 3). In males, as the frequency per minute of gestures produced increases, the probability of belonging to the group of late talkers decreases. For both males and females, the higher the gesture production at 12 months, the higher was the probability to be an early talker. As for the other variables, the probability values of belonging to a group was then explored for the low, medium, and high values of the gestural production at 12 months, ( Table 6).

**Table 6.** The probability of belonging to the early, typical al late talkers for low, medium, and high values of the variable gestural productions at 12 months.

	Early talkers	Typical talkers	Late talkers
Values of gestures < 25 ^th^ percentile			
Female	0.50	0.40	0.08
Male	0.27	0.10	0.62
Values of gestures 25 ^th^-75 ^th^ percentile			
Female	0.44	0.48	0.07
Male	0.26	0.13	0.59
Values of gestures > 75 ^th^ percentile			
Female	0.37	0.56	0.06
Male	0.26	0.18	0.55

3.3.4 The role of CDI actions and gestures

In relation to actions and gestures, ( Figure 4), results suggested that the higher the number of gestures and actions at 15 months, the higher the probability of being in the ETs, and the lower the probability of being in the LTs, with a comparable trend for males and females. The probability values of belonging to a group was explored for the medium and high values of the actions and gestures model at 15 months ( Table 7).

**Table 7.** The probability of belonging to the early, typical al late talkers for low, medium, and high values of the variable CDI Actions and Gestures at 15 months.

	Early talkers	Typical talkers	Late talkers
Values of CDI actions and gestures < 25 ^th^ percentile			
Female	0.66	0.02	0.30
Male	0.19	0.01	0.79
Values of CDI actions and gestures 25 ^th^-75 ^th^ percentile			
Female	0.56	0.36	0.06
Male	0.26	0.13	0.59
Values of CDI actions and gestures > 75 ^th^ percentile			
Female	0.13	0.85	0.00
Male	0.11	0.82	0.06

3.3.5 The role of CDI receptive vocabulary size

**Figure 6.**
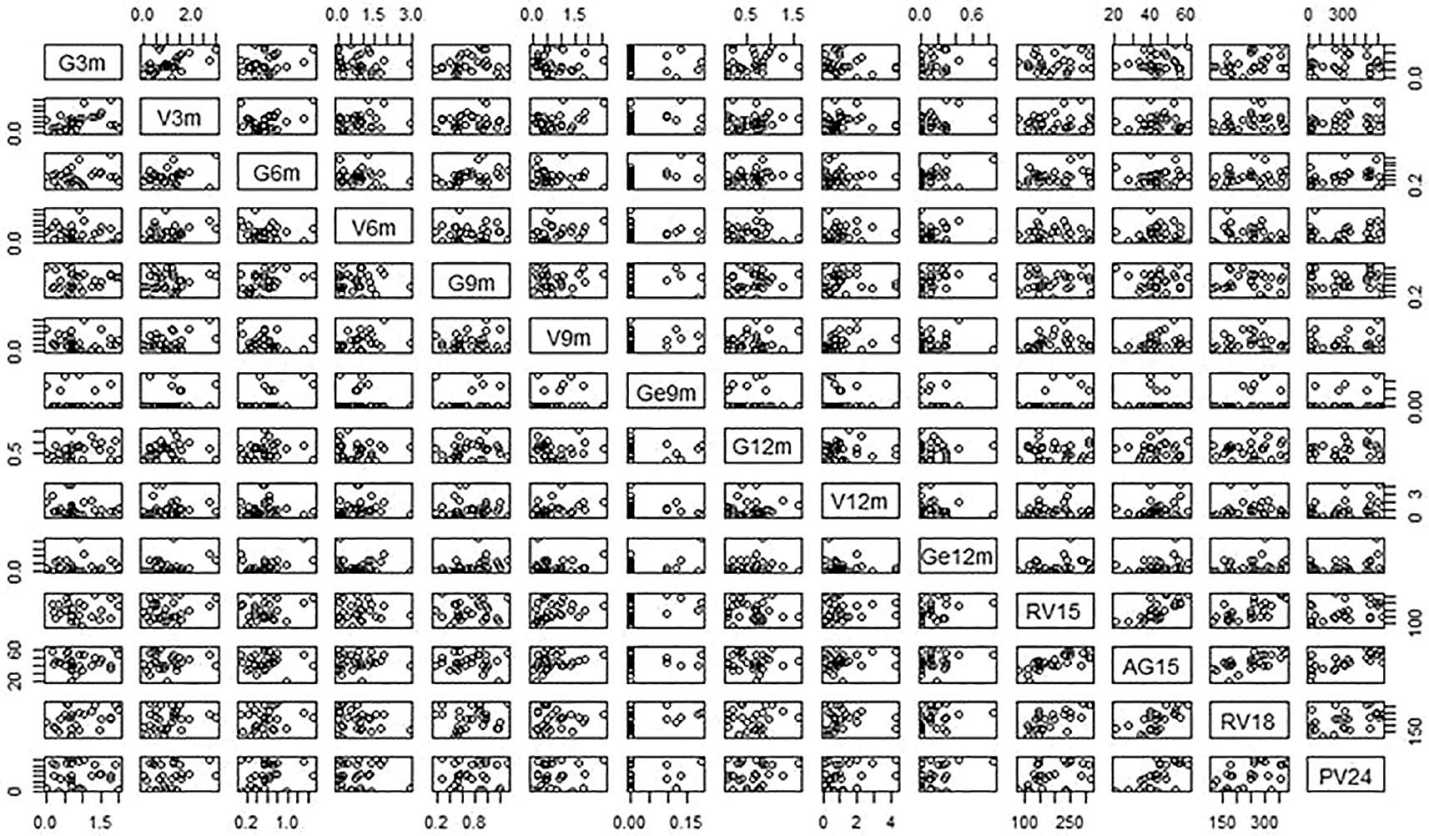
Plot variable correlation. Note: V is the abbreviation of Vocal at 3, 6, 9 and 12 months; G is the abbreviation of Gaze at 3, 6, 9 and 12 months, GE is the abbreviation of gestural production at 9 and 12 months, AG is the abbreviation of MB-CDI at 15 months, RV is the abbreviation of MB-CDI Receptive Vocabulary size at 15 and 18 months, and PV is the abbreviation of MB-CDI Productive Vocabulary size at 24 months.

Finally, we investigated the role of receptive vocabulary at 15 and 18 months. As reported in the plot of predicted probabilities ( Figure 5), as the receptive-vocabulary size increases, the probability of being ETs increases too, with a similar trend for both male and female, while the probability of belonging to TTs and LTs decreases. The probability analysis of receptive vocabulary size is in Table 8. The probabilities of belonging to the group for low, medium, and high values of the variables of interest were then explored, considering the mean of the receptive-vocabulary size at 15 and 18 months when possible, or only one time point if data were available for only one assessment phase ( Figure 6).

**Table 8.** The probability of belonging to the early, typical al late talkers for low, medium, and high values of the variable CDI Receptive vocabulary size at 15 and 18 months.

	Early Talkers	Typical Talkers	Late Talkers
Values of CDI receptive vocabulary size < 25 ^th^ percentile			
Female	0.63	0.15	0.21
Male	0.18	0.01	0.79
Values of CDI receptive vocabulary size 25 ^th^-75 ^th^ percentile			
Female	0.47	0.45	0.07
Male	0.30	0.10	0.59
Values of CDI receptive vocabulary size > 75 ^th^ percentile			
Female	0.21	0.77	0.01
Male	0.30	0.40	0.28

## 4. Discussion

The purpose of the study was to explore early predictors of the probability to be a late, typical, or early talker. In doing this, we investigated age points never considered in the prior literature. We specifically focused on a series of interactive behaviors, analyzed in the context of the parent-child interaction, as potential predictors. Moreover, we explored the contribution of gender. Our findings suggest that the roots of variability in the expressive vocabulary size, that can be reliably assessed when children are two years old, can be found very early in life, in infants as young as 3 months of age. More specifically, we found that vocal production at 3 months of age can comparatively be more able to predict subsequent language development than vocal productions later, and that the gaze at the caregiver’s face at 6 months can predict group membership better than gaze at the caregiver’s face later. Interestingly, even if based on a relatively small sample, the pattern of findings identified was consistent with the theoretical expectations. Specifically, that the higher was the frequency of the specific interactive behaviors considered, the higher was the likelihood to belong to an early talker vs. a typical or later talker’s condition. This study, although preliminary and explorative in nature, highlights the complexity that characterizes development. In line with what has been conceptualized as a “cognitive cascade”, in which elementary skills are the basis of more complex ones ( Rose, Feldman, Jankowski & Van Rossem, 2008), language development also follows a “cascade effect”, in which early communicative skills are the basis of more advanced abilities ( Fasolo, D’odorico, Costantini & Cassibba, 2010). Overall, our results emphasize that human language must be seen as structurally related to non-linguistic behaviors ( Vihman, Macken, Miller, Simmons & Miller, 1985). In fact, from the earliest months the newborn produces simple sounds ( Locke, 1989). Child’s vocal productions generate immediate and regular consequences in the social environment and gradually become more complex thanks to neurobiological maturation and the continuous interactions with the caregiver ( Desmarais, Sylvestre, Meyer, Bairati, & Rouleau, 2008). The exercise in prelinguistic productions expands the repertoire of sounds and sequences of sounds to which the first words are associated with ( Locke, 1989). Therefore, it is possible that at an early age the initial vocal production could have a “cascade effect” ( Fasolo et al., 2010) on the more complex competences expressed at two years. The gaze at caregiver’s face at 6 months is the variable that better predicts the later language outcome. Indeed, the selective attention to the social scene, such as the attention to the caregiver’s face, is positively correlated with language development ( Brooks & Meltzoff, 2005, 2008; Carpenter, Nagell, Tomasello, Butterworth & Moore, 1998). These findings suggest that the pre-linguistic production and the gaze at mother’s face, can be considered an expression of a “communicative instinct” that changes over time, and which unfolds in the face-to-face exchange between the infant and the caregiver ( Tomasello & Kruger, 1992). When approaching the first year of life, children begin to use their motor skills for social, thus producing representational and deictic gestures that are aimed at communicating intentions and meanings ( Aureli, Spinelli, Fasolo, Garito, Perucchini, & D’Odorico, 2017). In line with existing literature, our study underlies that the production of gestures at 12 and actions and gestures at 15 months is a predictor of the productive vocabulary size at two years ( Iverson & Goldin-Meadow, 2005). The different degree of use of actions and gestures may be representative of a different degree of maturity of symbolic function, which is the underlying ability of the language development process ( Bates et al., 1979). It is supposable that the competences, that children exercise with the production of actions and gestures during social interactions, associated with several developments in other areas (e.g., cognitive maturity) and parent’s responsiveness ( Aureli, Spinelli, Fasolo, Garito, Perucchini & D’Odorico, 2017; Tomasello & Kruger, 1992) can influence the ability to attribute symbolic autonomy to a word. This ability is considered by some authors as fundamental in determining the transition from the first to the second lexical phase which determines the rapid increase in the productive vocabulary size ( Bates et al., 1979). Regarding gender, consistent with data in the literature, our study found that gender appears to contribute to differences in language outcomes at 24 months (Huttenlocher, 1991). Specifically, females in our study seem to be more advantaged than males and were more likely to belong to the group of early or typical talkers, especially in the first year of life. It is important to specify that the conclusions on this outcome should be considered with caution. First, the imbalance between males and females is a question that does not allow us to generalize the data. Second, it is not possible to assume whether the differences are due to individual variables, such as gender, or environmental variables, such as the quantity or quality of input to which children are exposed.

## 5. Conclusions

The study investigates linguistic trajectories of children at the opposite side of language spectrum - late and early talkers – starting from 3 months. We used a longitudinal de-sign, and we adopted an observational method. However, findings should also be considered in the light of some limitations. First, even if the sample size was noteworthy given the longitudinal design and the use of observational and laboratory-based measures, for reaching more conclusive research findings, a bigger sample is needed. Stated this, it is important to note that our sample is comparable to that adopted in other studies on late and early talkers ( Fisher, 2017). Specifically, the percentage of late talkers we identified is consistent with empirical research and data reported in the international literature ( Bello, Olioso, Anghinoni, Gavalotti & Caselli, 2014; Horwitz, Irwin, Briggs-Gowan, Heenan, Mendoza & Carter, 2003; Zubrick, Taylor, Rice, Slegers, 2007). A second limit is related to the unbalanced gender distributions. However, the number of males and females that we identified in the group of early and late talkers is coherent with data reported in the literature about early gender differences, emphasizing a language advantage in females ( Doran, 1907; Nelson, 1973; Reznick & Goldfield, 1989). A third limit is that the interactive behaviors referring to the second year of life (CDI number of actions and gestures and CDI receptive-vocabulary size) were based exclusively on parent-report. In future studies, direct observational measures should be included at all time points, to evaluate interactive behaviors both during the first and second year of life. Finally, this study must be contextualized to the specific background in which the data have been collected, involving children from a single Country and with the same ethnic background. Future studies should consider the involvement of a larger group of children with different ethnic and socio-cultural backgrounds.

Notwithstanding these limitations, we believe that our study points out the importance of the identification of communicative predictors of the expressive vocabulary starting from very early in life. Our study points out potential theoretical and applied implications. From a theoretical perspective, the early identification of the roots of belonging to the group of late or early talkers, underlie the main role of this evolutionary period in future studies. On the other hand, from an applied point of view, our results specify and reinforce the importance of an early investigation in order to identify the predictors of vocabulary development prior to the age point at which lexical delay is usually identified.

The early identification of children that might be more at risk of a delay in language acquisition can enable access to early intervention and education programs that maximize positive child developmental outcomes across a variety of domain. Importantly, because all investigated communicative indices were observed in a dyadic context, these results suggest that the parent-child dyad could be the target of early intervention and prevention programs. All the interactive behaviors considered imply skills that promote interactive exchange, confirming once again that language develops primarily in the context of face-to-face interaction between the infant and the caregiver. A key question for future studies could be the investigation of the effects of the caregiver’s communicative and linguistic competencies on the children linguistic path.

## Ethics and consent

The study was approved by the ethical committee of the Department of Neuroscience Imaging and Clinical Science of the University of Chieti-Pescara (Ethical approval number: DNISC2962, 06.11.2019) and was conducted according to the American Psychological Association guidelines in accordance with the 1964 Helsinki Declaration.

A written informed consent (approved by the ethical committee) for participation in the study has been obtained by the mothers.

## Data Availability

Dryad: Early, typical, and late talkers: an exploratory study on predictors of language development in the first two years of life. https://doi.org/10.5061/dryad.wwpzgmsrb ( Logrieco, 2024a). This project contains the following underlying data:
• LateEarlyTypical_Talker.xlsx. Raw data file which includes the child’s data. • read_me_.docx. An explanatory document. Data are available under the terms of the Creative Commons Zero “No rights reserved” data waiver (CC0 1.0 Public domain dedication).
