# Supplementary material for: Early, typical, and late talkers: an exploratory study on predictors of language development in the first two years of life
Source: F1000Res. 2024 Jul 16;13:798. [Version 1] doi: 10.12688/f1000research.145763.1 (PMC11320034; doi:10.12688/f1000research.145763.1)
Supplement: Peer review history

# Reviewer report: Reviewer response for version 1

**DOI:** 10.5256/f1000research.159754.r309659

**Published:** 2024-08-12

**Authors:** Maria Chiara Pino

**Affiliations:** 1 University of L'Aquila, L’Aquila, Italy

Logriego et al.’s study represents an important contribution to research on the predictors of language development during the first two years of life. The subject matter is topical because the prevalence of late talkers and their difficulties in other domains might have increased due to the atypical constraints associated with the COVID-19 pandemic and widening economic and educational inequalities. The authors identify the interactive behaviours—between 3 and 18 months—that best predict early, typical, or late talkers (by the 24th month). The results show they can manifest as early as 3 months for vocal production, 6 months for facial gazing at the mother, and 12 months for gestural production.

              

 This well-written and well-designed study complements the emergent literature on how social cues produced by a child from 3 months can predict subsequent language outcomes (i.e., the breadth of productive vocabulary at 2 years).

              

 The theoretical discussion of the early markers (not only linguistic ones) and developmental trajectories of language is sound, although it would benefit from the inclusion of other papers on this topic (see below). A strong point of the study is the longitudinal design, which is based on data from 3, 6, 9, and 12-month-old infants.

 I would like to make the following suggestions:

  

- The principal aim of the study should be specified before the method section in the Abstract.
- The authors should specify whether the descriptive values differ between groups in Table 1 (notwithstanding the small sample size).
- The following studies might be cited regarding the state of the art in the field:

Caselli, M. C., Rinaldi, P., Stefanini, S., Volterra, V. Early action and gesture “vocabulary” and its relation with word comprehension and production. Child Dev. 2012, 83, 526–542.

 D’Amico, S., De Cagno, A. G., Levorato, M. C., Rossetto, T., Sansavini A. (2022). Il Disturbo Primario di          Linguaggio. Oltre la Consensus Conference. Trento: Erickson, ISBN: 9788859025900.

 Sansavini, A., Bello, A., Guarini, A., Savini, S., Stefanini, S., Caselli, M. C. Early development of gestures, object-   related-actions, word comprehension and word production and their relationships in Italian infants: A             longitudinal study. Gesture 2010, 10, 52–85.

 Sansavini, A., Favilla, M. E., Guasti, M. T., Marini. A., Millepiedi, S., Di Martino. M. V., Vecchi. S., Battajon. N.,     Bertolo, L., Capirci, O., Carretti, B., Colatei, M. P., Frioni, C., Marotta, L., Massa, S., Michelazzo, L.,      Pecini, C., Piazzalunga. S., Pieretti, M., Rinaldi, P., Salvadorini, R., Termine, C., Zuccarini, M., D’Amico. S.,         De Cagno, A. G., Levorato, M. C., Rossetto, T., Lorusso, M. L. Developmental Language Disorder: Early      Predictors, Age for the Diagnosis, and Diagnostic Tools. A Scoping Review. Brain Sci. 2021 May           17;11(5):654. doi: 10.3390/brainsci11050654. PMID:               34067874; PMCID: PMC8156743.

  

- The authors cite Rowe et al.’s (2020) important contribution to the field in their bibliography, but I cannot find a reference to it in the main text of the manuscript. They should consider their findings in light of Rowe et al.’s proposed classification.

 

- Finally: did the authors consider other factors that can affect language development, for example, a mother’s speech, preterm birth, nursery attendance, frequent otitis, and other relevant health conditions? These aspects can be seen as limits and possible future perspectives.

Is the work clearly and accurately presented and does it cite the current literature?

Partly

If applicable, is the statistical analysis and its interpretation appropriate?

Yes

Are all the source data underlying the results available to ensure full reproducibility?

No source data required

Is the study design appropriate and is the work technically sound?

Yes

Are the conclusions drawn adequately supported by the results?

Yes

Are sufficient details of methods and analysis provided to allow replication by others?

Yes

Reviewer Expertise:

- Study of developmental trajectoriesof socio-emotional skills in children with typical and atypical development.  -Linguistic and socio-cognitivedevelopment: relationship between cognitive and linguistic domains at different stages oflearning; predictive indices of development. -Positive adaptation in the lifecycle: multidimensional analysis of life styles and choices and adaptation tothe environment, both school and community, in adolescents and young adults. -Application of technology-basedinterventions to enhance social and emotional skills in children with atypicaldevelopment.     - Application of models and techniques characterising the study of socio-emotional skills. Studyof the relationship between reading and language development and the readingprocesses involved in the promotion of social-emotional, affective andrelational skills.

I confirm that I have read this submission and believe that I have an appropriate level of expertise to confirm that it is of an acceptable scientific standard.

## References

[1] : Early action and gesture "vocabulary" and its relation with word comprehension and production. *Child Dev* .2012;83(2) : 10.1111/j.1467-8624.2011.01727.x 526-42 10.1111/j.1467-8624.2011.01727.x 22304431

[2] : Primary Language Impairment: Beyond the Consensus Conference. *ITALY:Ericsson* .2021;

[3] : Early development of gestures, object-related-actions, word comprehension and word production, and their relationships in Italian infants. *Gesture* .2010;10(1) : 10.1075/gest.10.1.04san 52-85 10.1075/gest.10.1.04san

[4] : Developmental Language Disorder: Early Predictors, Age for the Diagnosis, and Diagnostic Tools. A Scoping Review. *Brain Sci* .2021;11(5) : 10.3390/brainsci11050654 10.3390/brainsci11050654 34067874 PMC8156743

# Reviewer report: Reviewer response for version 1

**DOI:** 10.5256/f1000research.159754.r304096

**Published:** 2024-08-09

**Authors:** Mara Morelli

**Affiliations:** 1 Universita degli Studi di Roma La Sapienza, Rome, Lazio, Italy

**Comments and Suggestions for Authors:** Thank you for the opportunity to review this manuscript. This interesting and needed study investigated predictors of vocabulary size that may help professionals to identify children's performance as late, typical, or early talkers. A strength of this study is the age points included. While most of the research has included children starting at ten months of age, this study investigated predictors as early as three months. Minor edits are needed.

**Participants**: In the theoretical introduction, the authors mention the fact that gender can impact language acquisition. How can the imbalance between males and females be justified in your sample?

**Participants**: Why were only mothers chosen for the dyad study? Work on fathers exists. It would be important to specify this choice in the problematic section.

Is the work clearly and accurately presented and does it cite the current literature?

Yes

If applicable, is the statistical analysis and its interpretation appropriate?

Yes

Are all the source data underlying the results available to ensure full reproducibility?

Yes

Is the study design appropriate and is the work technically sound?

Yes

Are the conclusions drawn adequately supported by the results?

Yes

Are sufficient details of methods and analysis provided to allow replication by others?

Yes

Reviewer Expertise:

Developmental psychology

I confirm that I have read this submission and believe that I have an appropriate level of expertise to confirm that it is of an acceptable scientific standard.
